# Contributions of Support Point Number to Mirror Assembly Thermal Sensitivity Control

**DOI:** 10.3390/s23041951

**Published:** 2023-02-09

**Authors:** Haixing Li, Hongwen Zhang, Yalin Ding, Jichao Zhang, Yuqi Cai

**Affiliations:** 1 Changchun Institute of Optics, Fine Mechanics and Physics, Chinese Academy of Sciences, Changchun 130033, China; 2 Department of Basic Education, Harbin Institute of Technology, Harbin 150001, China

**Keywords:** mirror mount, support system, thermal sensitivity, temperature variation

## Abstract

Due to the extreme environmental temperature variations, solutions that enable ultra-low thermal sensitivity in a mirror assembly are crucial for high-performance aerial optical imaging sensors (AOIS). Strategies such as the elimination of the coefficient of thermal expansion (CTE) mismatch and the employment of a flexure connection at the interface cannot be simply duplicated for the application involved, demanding specific design constraints. The contributions of support point number to the surface thermal sensitivity reduction and support stiffness improvement have been studied. A synthetic six-point support system that integrates equally spaced multiple ultra-low radial stiffness mirror flexure units and assembly external interface flexure units has been demonstrated on a 260 mm apertured annular mirror that involves significant CTE mismatch and demanding support stiffness constraint. The surface deformation RMS, due to the 35 °C temperature variation, is 16.7 nm.

## 1. Introduction

With variations of seasons, operating locations, flight altitudes, and weather conditions, aerial optical imaging sensors (AOIS) endure extreme temperature variations that introduce a large gradient, fast rate, and significant variation. Under some extreme conditions of typical flight operation, environmental temperature change can reach nearly 100 ℃ in dozens of minutes from the ground level to the flight altitude [1,2,3]. Compared with the laboratory ambient conditions, at which the AOIS is integrated and calibrated, temperature variation under aerial working conditions reaches dozens of degrees Celsius. Even though various thermal management measures have been employed [4,5,6,7], the AOIS internal temperature variation is still remarkable. With improvements in AOIS system performance requirements and mechanical structure compactness levels, the frequent selection and massive application of cost-effective low-density materials, such as aluminum alloy, etc., for light-weight and structure miniaturization purposes causes significant coefficient of thermal expansion (CTE) mismatch between the mirror assembly and AOIS main structure.

Due to the coexistence of large temperature variations and significant CTE mismatch between the mirror assembly and the external support structure, the large magnitude of thermal coupled mechanical load (TCML) significantly stresses the mirror assembly [8]. The key to reducing surface thermal sensitivity is to reduce the TCML sensitivity at the mirror support point. Aspects that influence TCML sensitivity include: (1) connection stiffness at interface; (2) CTE mismatch; and (3) size of the area encircled by support points. Theoretically, reduction of either of the three aspects contributes to the reduction in mirror surface thermal sensitivity.

For the purpose of high precision optical alignment, support stiffness is also one of the key concerns during the mirror assembly design process. As for high performance AOIS, the surface decenter and the surface tilt due to gravity are usually needed to be micron level and arc second level, respectively. Because the reduction in surface thermal deformation sensitivity partially correlates with the improvement in support stiffness, the implementation of an ultra-low surface thermal sensitivity mirror assembly that retains high support stiffness is crucial and extremely challenging for the high performance AOIS. Influences of the mirror support scheme on surface thermal sensitivity and mechanical stiffness have been previously reported by David Chin [8]. In the literature, a tangential flexure strap has been employed on a three-point side-supported circular mirror to reduce the surface thermal sensitivity. Since then, three-point support systems that employ various flexures have been widely used [9,10,11,12,13,14,15,16,17]. This group of mirror mount systems mainly uses radial flexure to reduce the TCML sensitivity due to CTE mismatch across the interface [15,16,17]. Some of them also arrange flexures at the mirror assembly external interface to filter the external thermal and mechanical disturbances [14]. Due to the stiffness coupling effect at the interface, it is extremely difficult to compensate for the excessive radial stiffness reduction that is derived from extensive temperature suitability simply by the excessive tangential stiffness increase at the mirror support point.

The reduction or elimination of CTE mismatch, which is simple and much more straightforward, has been widely used. Typically, all-SiC optical modules have also been reported [18,19,20]. This type of optomechanical system can significantly reduce the mirror surface thermal deformation sensitivity, while still maintaining excellent support stiffness. However, the all SiC configuration will lead to remarkable increases in financial and project time costs. A center-mounted mirror assembly used on the extreme-ultraviolet camera onboard the Chang’e lunar lander has been reported by Li, Z. et al. [21]. Zerodur and Invar are respectively selected for the mirror blank and the back plate. Multi-point side support schemes that employ radial flexure for various purposes, such as local stress reduction, thermal and mechanical shock isolation, and thermal sensitivity reduction, have also been reported [22,23,24]. However, studies on the feasibility of an ultra-low thermal sensitivity mirror assembly that can accommodate significant CTE mismatch and still retain high support stiffness, have not been reported.

To find a low-cost solution to the implementation of an ultra-low thermal sensitivity mirror assembly that retains high support stiffness and can also accommodate significant CTE mismatch for high performance AOIS, we concentrated on the contributions of support point number to surface thermal deformation sensitivity reduction and support stiffness improvement. In this paper, in Section 2, we summarize the influencing factors of mirror surface thermal deformation and the strategies for reducing surface thermal deformation sensitivity. The TCML and the related control strategies, respectively, are introduced and analyzed in Section 3. Contributions of support point number to TCML control at the mirror support point, as well as surface thermal deformation sensitivity reduction, are discussed in Section 4. The design process and the calculation of the stiffness vector at the mirror support point, respectively, are summarized and discussed in Section 5. A six-point side support system that employs equally spaced multiple ultra-low radial stiffness mirror flexure (MF) units and assembly external interface flexure (AEIF) units is demonstrated in the Section 6. The proposed scheme provides a viable solution for the implementation of an ultra-low thermal sensitivity mirror support system suitable for small- to medium-sized mirrors that involves significant CTE mismatch and demanding support stiffness requirements.

## 2. Aspects of Surface Thermal Deformation

**Figure 1:**
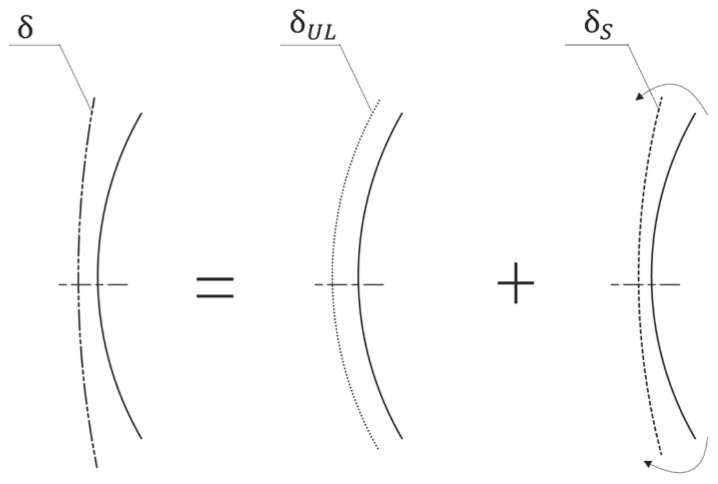
Illustration of surface deformation due to temperature variation.

Illustrated as Figure 1, surface thermal deformation δ can be viewed as the combination of the linear thermal expansion $\delta_{UL}$, due to the freeform expansion, and the elastic deformation $\delta_{S}$, due to the TCML exerted at the mirror support point, and is expressed as,

(1) $$\delta =\delta_{UL}+\delta_{S}$$

The linear thermal expansion $\delta_{UL}$ that is directly proportional to the temperature variation ΔT, the mirror CTE $a_{M}$, and the surface radius $R_{0}$, uses the following formulation:

(2) $$\delta_{UL}=\Delta Ta_{M}R_{0}$$

The TCML induced surface elastic deformation $\delta_{S}$, which is inversely proportional to the mirror rigidity $k_{M}$ and directly proportional to the TCML magnitude $F_{T}$ exerted at mirror support point, is expressed as,

(3) $$\delta_{S}=f(k_{M},F_{T})$$

Methods to reduce surface thermal deformation sensitivity include: (1) the selection of ultra-low CTE mirror material, (2) the improvement of mirror rigidity, and (3) the reduction of TCML sensitivity at the mirror support point. The selection of ultra-low CTE materials is the key to controlling surface linear thermal expansion sensitivity, particularly for applications involving large temperature variations. Mirror rigidity, which is the intrinsic property that determines the surface elastic deformation quantity due to unit external mechanical stress, determines the mirror assembly temperature variation suitability. Under any circumstances, a high rigidity mirror blank is crucial for the achievement of large temperature variation suitability. The coexistence of a large temperature variation and significant CTE mismatch between the mirror assembly and external structure generates extreme challenges for the reduction of TCML sensitivity at the mirror support point.

## 3. The TCML at the Mirror Support Point and Sensitivity Control

### 3.1. Thermal Coupled Mechanical Load

**Figure 2:**
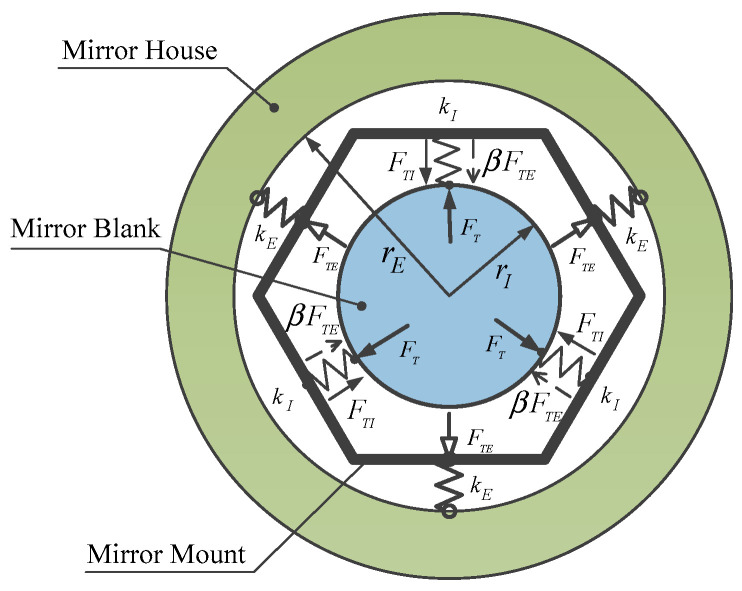
Illustration of TCML acting on the mirror.

A simplified analysis model is illustrated as Figure 2. The mirror assembly that consists of a mirror blank, the equally spaced flexure connections, and the mirror mount, is assumed to be suspended in the plane and externally connected with the mirror house by three equally spaced equal-stiffness springs.

Under uniform temperature variation, the CTE mismatches that occur across the mirror assembly internal interface and the external interface, respectively, cause TCMLs at different hierarchies of the connecting points. The TCML that is due to the CTE mismatch between the mirror blank and the mirror mount is the mirror assembly internal interaction, and it is called the internal thermal coupled mechanical load (ITCML). The TCML that is due to the CTE mismatch between the mirror mount and the external mirror house is the mirror assembly external interaction, and it is called the external thermal coupled mechanical load (ETCML). The ITCML acts at the mirror support point directly; however, the ETCML is attenuated by the mirror mount and acts at the mirror support point indirectly. The total TCML magnitude at each mirror support point can be expressed as,

(4) $$F_{T}=F_{TI}+\beta F_{TE}$$

where $F_{TI}$, $F_{TE}$, and β are assumed to be the ITCML magnitude, the ETCML magnitude, and the ETCML transfer factor, respectively. The ETCMLs that act at the mirror assembly external interfaces stress the mirror mount, cause forced displacement at the connecting point of the flexure that connects the mirror mount and the mechanical interface bonded with the mirror support point. Due to the protecting effect of the mirror mount on the mirror blank, the majority of the ETCML is attenuated by the elastic deformation of the mirror mount. The product of the ETCML magnitude at the external interface and the ETCML transfer factor can be viewed as the portion of the ETCML that is transferred by the back plate of the mirror mount from the external interface to the connecting point at the internal flexure, and it is balanced by the local elastic deformation of the flexure at the mirror support point.

Either the ITCML sensitivity at the mirror support point or the ETCML sensitivity at mirror assembly external interface are governed by: (1) the CTE mismatch of the adjacent two parts, (2) the connecting stiffness at interface, and (3) the radius of the area encircled by the interfaces. The magnitudes of the two can be expressed as,

(5) $$F_{TI}=(\alpha_{M}-\alpha_{BP})k_{I}r_{I}\Delta T$$

(6) $$F_{TE}=(\alpha_{E}-\alpha_{BP})k_{E}r_{E}\Delta T$$

where $\alpha_{M}$, $\alpha_{E}$, and $\alpha_{BP}$ are, respectively, CTEs of the mirror blank, the external mirror house, and the mount structure; $k_{I}$ and $k_{E}$ are, respectively, the connecting stiffnesses at the mirror support point and the mirror assembly external interface; $r_{I}$ and $r_{E}$ are the radii of the two areas that are, respectively, encircled by the mirror support points and the external interfaces.

### 3.2. Summary of TCML Sensitivity Control

Control strategies of the ITCML sensitivity at the mirror support point and the ETCML sensitivity at the mirror assembly external interface can be summarized as: (1) the elimination of CTE mismatch across the interface, (2) the minimization of the area encircled by the interfaces, and (3) the reduction of connecting stiffness at the interface. Each of the aspects makes a similar contribution to the TCML sensitivity reduction. If the AOIS is ideally made of one type of material, or materials which have the same CTEs, the equipment will be immune to TCML. Due to some other constraints, such as mass, cost, material availability, process maturity, etc., the straightforward elimination of CTE mismatch that contributes significantly to the TCML sensitivity control is theoretically viable, but practically less feasible for implementation to the entire AOIS. The minimization of the area encircled by interfaces which are constrained by some other requirements, such as support stiffness, fastening security, mirror size, etc., also contributes to the reduction in TCML sensitivity.

The reduction of connecting stiffness at the interface is a widely used method for controlling TCML sensitivity. For the applications involving significant CTE mismatch and demanding support stiffness requirements, the excessive reduction in connecting stiffness that is derived from extreme temperature variation suitability will cause the reduction in support stiffness and generate some drawbacks to the engineering implementation, particularly for the support schemes that only use a few support points. Theoretically, the increase in connecting points contributes to the improvement of support stiffness for the entire mirror support system and therefore, has some effects on the reduction in connection stiffness at each of the mirror support points.

Moreover, improving the attenuation effect of the mirror mount, which usually involves the optimizations of the mount structure stiffness and the load path configuration, can also effectively attenuate the disturbance of the ETCML exerted at the mirror assembly external interface.

## 4. Contributions of the Support Point Number

Under the constraint of the mirror displacement due to gravity, contributions of the support point number to the radial support stiffness and TCML induced surface deformation sensitivity have been studied. For simplification purposes, the mirror is assumed to be suspended in the plane by multiple equally spaced equal-stiffness springs that are arranged symmetrically according to the gravity direction, and only the radial stiffness of the spring connection is considered.

### 4.1. Contribution to Connecting Stiffness Reduction

**Figure 3:**
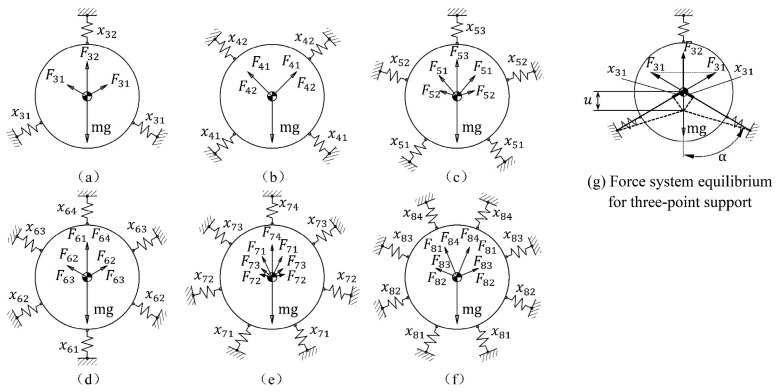
Support point configurations and the force systems for the multi-point support mode, under gravity along the radial direction. Mirror gravity is denoted by mg. $K_{Ri}$, $x_{ij}$, and $F_{ij}$ are spring stiffness, spring deflection, and corresponding spring force, respectively, for the support mode whose support point number is equal to i(i=3,4,5,6,7,8).

Support modes whose support point numbers vary from 3 to 8 have been studied. The typical configurations and the force systems are illustrated as Figure 3.

Based on the three-point support mode (see Figure 3g), the spring forces corresponding to typical support points are expressed as,

(7) $$F_{31}=K_{R3}x_{31}=K_{R3}u\cos\alpha$$

(8) $$F_{32}=K_{R3}x_{32}=K_{R3}u$$

where u is the mirror displacement due to gravity, and the force system equilibrium equation is expressed as,

(9) $$\begin{array}{l} mg=F_{32}+2F_{31}\cos\alpha \\ \;=K_{R3}u(1+2\cos^{2}(60^{\circ }))\\ \;=1.5K_{R3}u \end{array}$$

Spring stiffness that fulfils the equilibrium condition for the three-point support mode is expressed as,

(10) $$K_{R3}=\frac{1}{1.5}\frac{mg}{u}$$

Support radial stiffness that fulfils the equilibrium condition of gravity equals the combination of all spring forces along the typical direction for different support point number configurations, which can be described as the product of mg/u by the multipliers that are listed in Table 1.

### 4.2. Contribution to Surface Deformation Sensitivity

**Figure 4:**
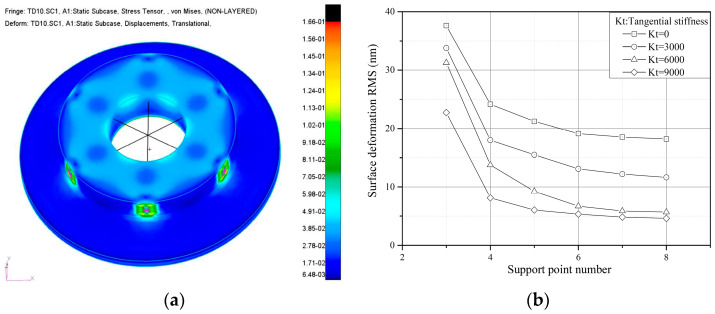
(**a**) FEA model, (**b**) Surface RMS vs. support point number. FEA model and TCML induced surface deformation sensitivity curve.

Based on the radial support stiffness requirement, which equals 1 micron side displacement due to gravity, the effects of the support point number on TCML-induced surface deformation sensitivity have been studied on an annular mirror. Surface thermal deformation due to TCML caused by a 10 °C temperature variation has been evaluated. The FEA model and curves of surface deformation RMS value vs. support point number under typical tangential stiffness are illustrated, respectively, in Figure 4a,b.

The results indicate that:

(a) Ideally, the radial support stiffness that balances with the mirror displacement due to gravity is inversely proportional to the support point number. Even though only the radial support stiffness is considered, the TCML-induced surface deformation sensitivity reduces dramatically with the increase in the support point number.
(b) Under the given mirror displacement constraints resulting from gravity, tangential stiffness at each support point contributes to the further reduction in radial support stiffness. For the same support point number configuration, TCML induced surface deformation sensitivity can be further reduced with increases in the tangential stiffness at each support point.

## 5. Theoretical Design of the Mirror Mount System

### 5.1. Design Process

Numerous research works have reported mirror mount system designs from aspects such as light-weight mirror optimization, support location optimization, flexure design, system performance evaluation, etc. [25,26,27,28]. Some research works have reported the mirror mount system design at the systematical engineering level [29].

**Figure 5:**
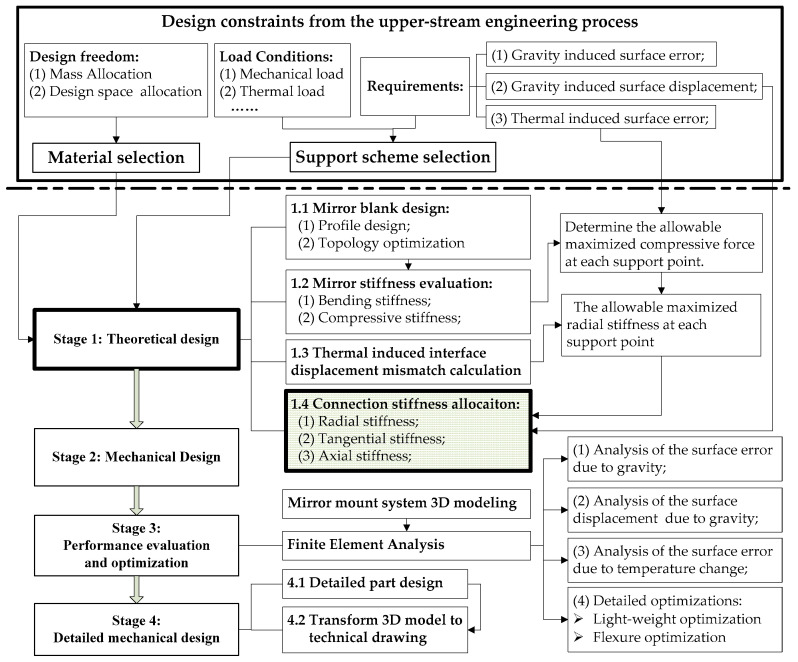
Mirror mount system design process.

**Figure 6:**
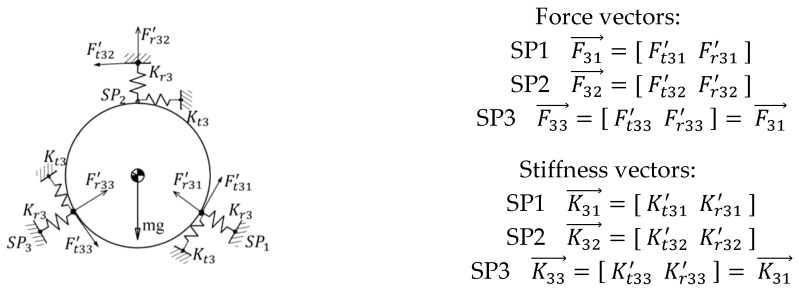
Illustration of the support vectors for the exemplified three-point support scheme.

The mirror mount system design process is illustrated in Figure 5; the key stages include: (1) the theoretical design, (2) the mechanical design, (3) the performance evaluation and optimization, and (4) the detailed mechanical design. The main objectives at the theoretical design stage are summarized as the following:

(a) Obtain an initially optimized mirror profile.
  Thickness of the mirror blank can be determined according to the material configuration and the allocated geometric design space. For large temperature variation suitability, a relatively thicker mirror blank is recommended. The distribution of the support points can be optimized under the constraint that the surface deformation error due to gravity along the axial direction is minimized.
(b) Determine the maximum allowable compressive force at the mirror support point.
  Based on the initially optimized mirror profile, the maximum allowable compressive force can be evaluated by the FEA on the simplified mirror assembly model (see Section 4.2).

(c) Calculate the thermally induced interface displacement mismatch.
  The radius of the support point distribution circle (see the $r_{I}$ in Equation (5)), the CTE mismatch between the mirror and the mount structure (see the $\left(\alpha_{M}-\alpha_{BP}\right)$ in Equation (5), and the considered temperature change (see the ΔT in Equation (5)), are required for this calculation.

(d) Obtain an initial radial stiffness that equals the required lateral support stiffness.
  The required lateral support stiffness (see the mg/u in Equation (10)) equals the mirror gravity divided by the allowable surface lateral displacement. The required radial stiffness (exemplified by the $K_{R3}$ in Equation (10)) equals to the required lateral support stiffness can be calculated by using the simplified model, as described in Section 4.1.

(e) Calculate components of the optimized stiffness vector at the mirror support point.
  Generally, the stiffness vector at each support point consists of an axial component, a radial component, and a tangential component (see Figure 6). For the mirror assembly that has high axis-symmetric properties, the axial component of the stiffness vector at support point can be solved in a relative independent manner. To simplify the illustration, consider the simplified two-element stiffness vector that consists of the radial component and the tangential component under high symmetric load conditions in-plane. Because only one equilibrium equation exists, a fixed solution to the stiffness vector that contains multiple components does not exist. Optimization is needed to calculate the stiffness vector.

### 5.2. Optimization of the Stiffness Vector at Mirror Support Point

**Figure 7:**
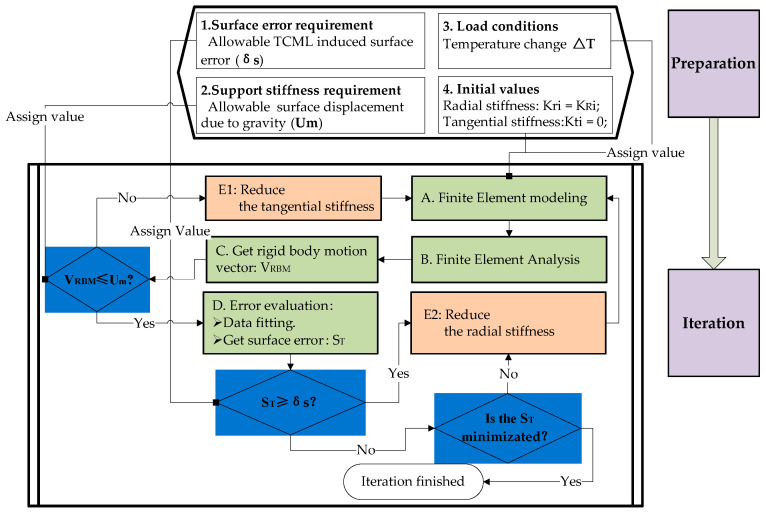
Flowchart of the optimization of the stiffness vector.

After the data preparation at the upper stream, optimization of the stiffness vector at the mirror support point can be implemented by following the flowchart shown in Figure 7. As we know, the key to reducing surface thermal sensitivity is reducing the radial stiffness at the mirror support point. The tangential stiffness (see the exemplified $K_{t3}$ in Figure 6) at the mirror support point also contributes to the lateral support stiffness of the mirror mount. Thus, the radial component of the stiffness vector (see the exemplified $K_{r3}$ in Figure 6) can be optimized downward from an upper initial value that is calculated in step d) (see Section 5.1). The tangential component of the stiffness vector should be optimized upward from a lower initial value that equals zero. In the process, the design objective is the minimization of the surface thermal sensitivity.

## 6. Demonstration

### 6.1. Requirements

During the optomechanical system engineering process of a typical high-performance AOIS, the performance of the primary mirror assembly has been allocated from the top system engineering process. The mirror surface thermal deformation RMS value due to a 35 °C temperature variation for a 260 mm apertured annular mirror used in a typical AOIS is required to be no larger than λ/35 (λ = 632.8 nm). The mirror assembly is directly connected with the aluminum external mirror house. The requirements are specified in Table 2.

### 6.2. Proposed Support System

**Figure 8:**
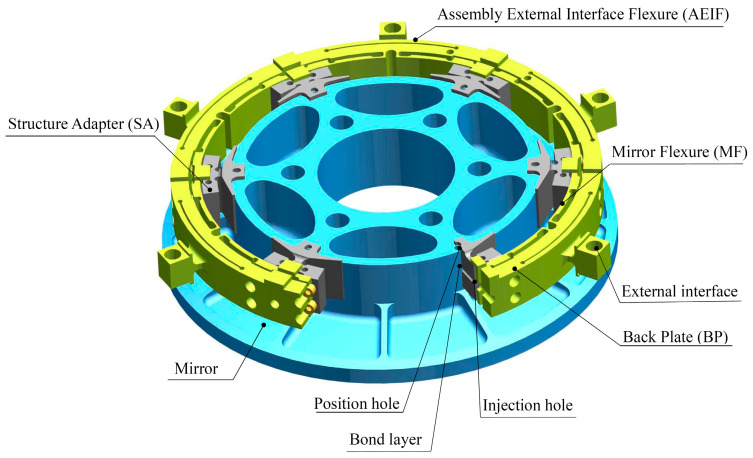
Mechanical structure of the mirror support system.

Based on the six-point side support mode, a synthetic support system that consists of six equally spaced Invar-made structure adapters (SAs) and one TC4-made BP that integrates six equally spaced MF units at the inner layer, AEIF units at outer layer, and assembly external interfaces at each AEIF center, is proposed, as shown in Figure 8. Each SA is bonded with the mirror blank at the support point and bolted to MF in the center. The mechanical properties of the materials used in the proposed mirror assembly are listed in Table 3.

The MF of the support system is featured, with an ultra-low radial stiffness that enables an ultra-low response to the radial forced displacements due to the mirror assembly internal thermal expansion mismatch and the ETCML-induced BP deformation, as well as low rotational stiffness that enables an ultra-low torque sensitivity at the mirror support point. The ETCML sensitivity at each external interface has been effectively reduced by the arc-shaped AEIF. Support stiffness has been compensated for by the increased number of external interfaces. The load path from each external interface to the adjacent MF junction with BP middle layer has been deliberately designed. Thus, very limited radial displacement due to ETCML is produced at each MF junction.

Fabrication of the proposed design conception involves several ordinary machining processes (at the earlier stage), wire electrical discharge machining operation (at the later stage), and a precision synthetic machining operation (at the final stage). The final stage precision synthetic machining is intended to achieve the coaxiality and the cylindricity of the six SA inner cylindrical faces, the fitting tolerance between the six SA inner cylindrical faces and the mirror side, and the coplanarity of the six small planes that are respectively settled on each SA and used for axial positioning of the mirror. At the assembling stage, the proposed support system can be easily positioned with the mirror along the radial direction and the axial direction respectively, and tangentially positioned with the mirror by plugging a pin into one of the six position holes that are settled on the mirror back and the SA ends, respectively. After the positioning process, a low expansion adhesive is injected into each bond area settled between the mirror side and each SA through the injection hole settled in the BP. After completing the adhesive cure process, a bond layer is formed at each support point of the mirror.

### 6.3. Discussions

#### 6.3.1. Mirror Topological Optimization

During the implementation of the mirror mount system, the evaluation of the surface sensitivity to the thermal or mechanical disturbances is the core concern. The framework that can integrate information streams from three groups of analysis tools: FEA, optimization, and optical performance evaluation, plays the key role. The integrated optomechanical analysis system was proposed by Doyle, K. B., et al. [30]. As for the mirror topological optimization at the earlier stage, a simplified FE model can be implemented. Under most situations at which the mirror has high symmetric profiles [31], we can conduct the optimization in a simplified manner.

**Figure 9:**
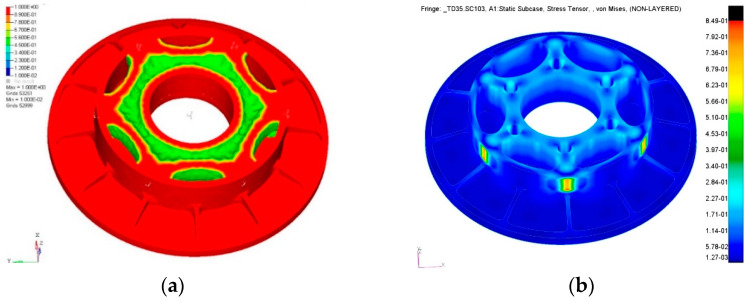
(**a**) Topological optimization result. (**b**) Stress contour plot. Stress contour plot of the mirror subjected to TCML.

For large temperature variation suitability, a relatively smaller diameter to thickness ratio that enables the excellent bending stiffness of the mirror blank is selected. The weight of the mirror has been lightened by topological optimization. The area between the outer periphery and central perforation side has been defined as the design space. After topological optimization, the hollowed mirror structure that consists of six equally spaced arch ribs, each sequentially connected to one support point, a central perforation wall, and the next adjacent support point, is obtained. The mirror geometric model was rebuilt by referring to the topological optimization results (Figure 9a). The stress contour plot of the finalized mirror due to 35 °C temperature variation is shown in Figure 9b.

#### 6.3.2. Ultra-Low Radial Stiffness MF

**Figure 10:**
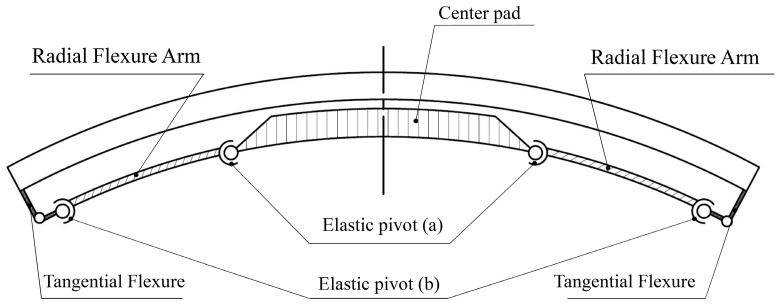
Multi-link mechanism of MF.

As basic unit of the mirror support system, the proposed MF as a multi-link mechanism, consists, respectively, of one movable center pad that connects each mirror support point sequentially via the bolted SA and the bond layer, two axially rotatable radial flexure arms, and tangential flexures which are symmetrically arranged (illustrated in Figure 10). Elastic pivots are arranged at the two ends of each radial flexure arm. The combination of an axially rotatable radial flexure arm and a tangential flexure enables the center pad translation degree of freedom along the radial direction. This configuration can realize not only ultra-low radial stiffness, but also very low axial torsional stiffness at each support point. Consequently, TCML sensitivity to temperature variation, as well as local axial torque sensitivity to assembly factors, can be significantly reduced.

#### 6.3.3. Effects of AEIF on ETCML Control

**Figure 11:**
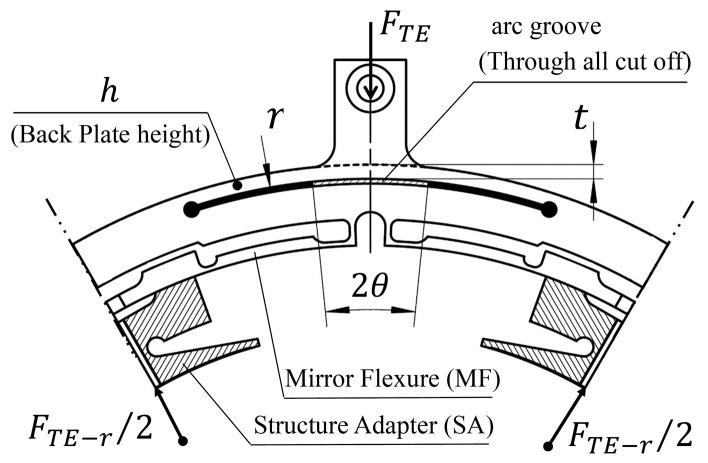
Parameters of the proposed AEIF.

To effectively improve the external disturbance attenuation effect of the support system and obtain an ultra-low response at the mirror support point, the AEIF that is used to reduce ETCML sensitivity at the external interface has been parameterized. The main design parameters of the AEIF are shown in Figure 11.

**Figure 12:**
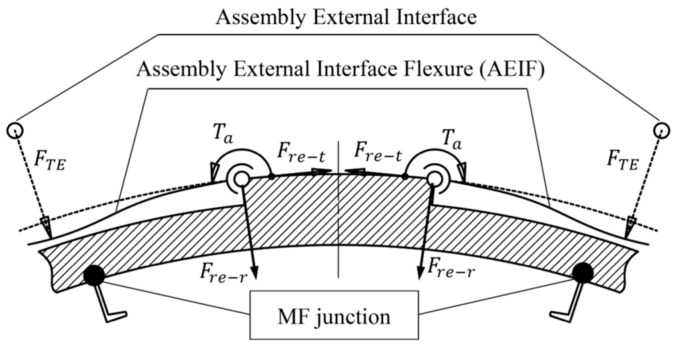
ETCML-induced AEIF deflection and equivalent load components at the AEIF end pivot.

As illustrated in Figure 12, a compressive ETCML exerted at each external interface, causing deflection, can be transformed to the two ends of each AEIF, and can be equivalently decomposed into three components: the radial force ($F_{re-r}$), the tangential force ($F_{re-t}$), and the axial torque ($T_{a}$). The tangential force pair mainly compresses the section that connects the two adjacent AEIFs along the circumferential direction. The radial force and axial torque locally deform the AEIF and bend the BP middle layer, causing the forced radial displacement at the MF junction. The ETCML-equivalent three transformed components at each AEIF end are expressed as,

(11) $$\begin{array}{l} \;T_{a}=0.5F_{TE}(r+0.5t)\sin\theta \\ F_{re-r}=0.5F_{TE}\cos\theta \\ F_{re-t}=0.5F_{TE}\sin\theta \end{array}$$

where θ, *t*, and *r* are, respectively, the groove semi-arc angle, the AEIF radial thickness, and the groove outside radius. The AEIF radial stiffness that influences ETCML sensitivity is mainly determined by the variables *t* and θ. Moreover, the load path length that influences the attenuation effect of the ETCML equivalent tangential force pair at each circumferentially compressed section, and thereby, the ETCML transfer factor β, is mainly determined by variable θ.

**Figure 13:**
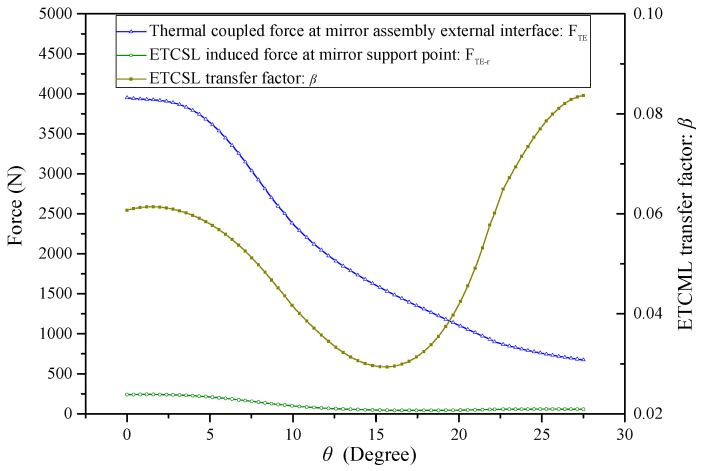
Relationships between the AEIF semi-arc angle (θ) and the ETCML ($F_{TE}$ ), the ETCML induced reaction force ($F_{TE-r}$ ), and the ETCML transfer factor (β ).

Effects of the groove semi-arc angle on the ETCML magnitude at the external interface, the ETCML induced reaction force at the mirror support point, and the ETCML transfer factor have been evaluated using FEA. The BP is assumed to be externally connected with the thermally expandable rigid SH and internally fixed at each MF center pad. The ETCML, and the ETCML-induced reaction force due to 35 °C temperature variation and CTE mismatch of aluminum-made SH and titanium-made BP, are investigated. Relationships between the groove semi-arc angle (θ) and the ETCML ($F_{TE}$), the ETCML induced reaction force ($F_{TE-r}$), and the ETCML transfer factor (β) are illustrated in Figure 13.

With an increase in the groove semi-arc angle, the AEIF radial stiffness and the involved ETCML sensitivity decrease dramatically. Moreover, the effects introduced to the load path from the external interface to the MF junction are summarized as follows:

(a) The ETCML equivalent radial force that compresses (or stretches) the BP middle layer along the radial direction decreases, according to the cosine law of the groove semi-arc angle.
(b) The increase in the load path length due to the groove semi-arc angle increase simultaneously causes the BP compressive stiffness reduction.
(c) The rigidity of the section that is deformed by the ETCML equivalent tangential force pair (see $F_{re-t}$ in Figure 12) and the axial torque pair (see $T_{a}$ in Figure 12) is improved.

When $\theta =0^{\circ }$, the MF compliance and the BP compressive stiffness play key roles in the attenuation of the ETCML. Due to the significant magnitude of the ETCML, the ETCML-induced reaction force at each mirror support point is still considerable. Under this situation, the ETCML transfer factor β=0.061.

When θ reaches nearly one-half of the angular separation between the two neighboring assembly external interfaces, the ETCML transfer factor reaches the valley level ($\beta_{\min}=0.028$ @ $\theta =15.24^{\circ }$). When $\theta =17.44^{\circ }$, the ETCML-induced reaction force reaches the minimum.

When the θ further increases and is greater than $17.44^{\circ }$, the AEIF radial stiffness decreases. The BP compressive stiffness is also reduced, and more forced radial displacement generates at the junction between the MF and the BP middle layer. The ETCML-induced reaction force at the mirror support point increases, to some extent. The ETCML transfer factor increases rapidly.

### 6.4. Performance Evaluation

**Figure 14:**
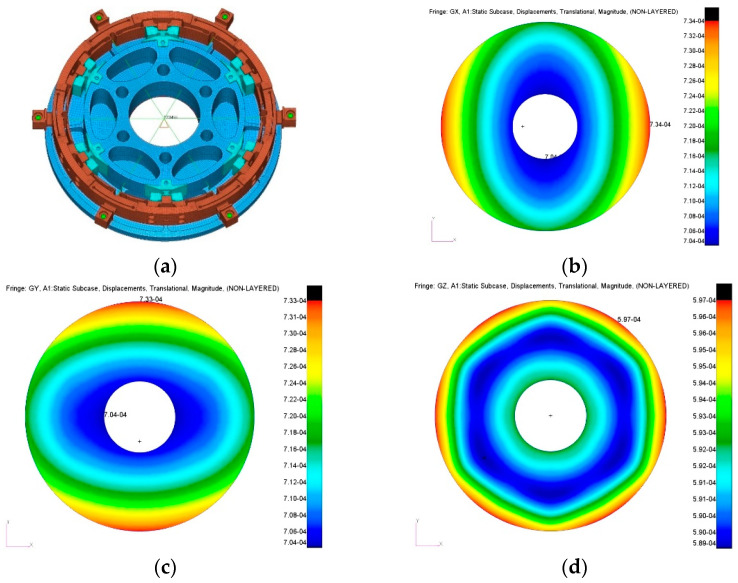
(**a**) FE model of the mirror assembly. (**b**) 1 g gravity along X direction. (**c**) 1 g gravity along Y direction. (**d**) 1 g gravity along Z direction. Mirror assembly FE model and surface displacement contour plot due to gravity.

The widely used optomechanical integrated analysis and optimization are crucial for the mirror assembly performance evaluation at the final stage [32,33,34,35]. To conservatively estimate thermal sensitivity of the mirror assembly at the FEA evaluation stage, the SH mechanical interface that is used to mount the mirror assembly is assumed to have infinitely large connection stiffness, and it can also thermally expand along radial direction. The SH is simplified to a six-arm rigid spider, whose central node is set at the mirror assembly center and each branch end node connects with one mirror assembly external interface. The fixed boundary condition is set on the central node. The mirror surface responses due to gravity along the X, Y, Z direction, and the 35 °C temperature variation, have been analyzed. The FE model and the surface displacement counterplot is shown in Figure 14.

**Figure 15:**
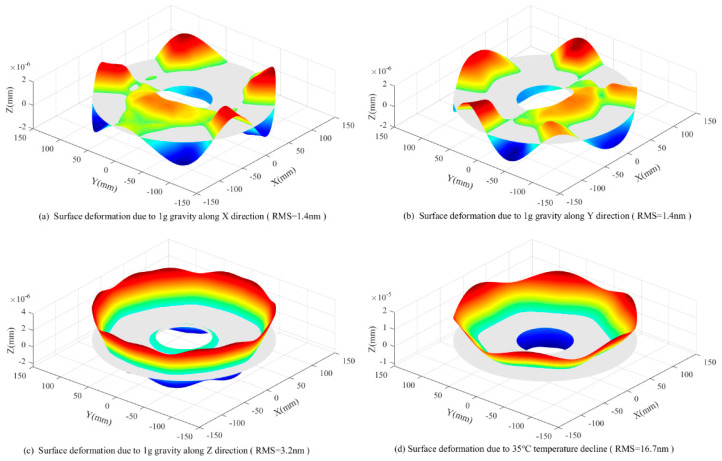
Surface deformation map.

Maps of the surface deformation after the removal of rigid body displacement are shown in Figure 15. The surface RMS value due to 35 °C temperature variation is 16.7 nm.

## 7. Conclusions

The aspects involved in reducing the surface thermal deformation sensitivity for the mirror assembly that involves the demanding constraints of significant CTE mismatch and high support stiffness requirements have been summarized. The contributions of the support point number to surface thermal sensitivity reduction and support stiffness improvement have also been studied.

For a typical AOIS that involves significant CTE mismatch at the mirror assembly external interface, a six-point support system that employs ultra-low radial stiffness at each of the mirror support points and a dedicated AEIF at each of the mirror assembly external interfaces has been demonstrated on a 260 mm apertured annular mirror that involves the demanding constraints of a 35 °C temperature variation suitability and a 1 micron surface displacement due to gravity. The surface RMS value due to the 35 °C temperature variation reaches 16.7 nm. Compared with the three-point support scheme, the proposed solution shows great improvements, not only in the aspect of temperature variation suitability, but also in the aspect of large CTE mismatch suitability.

The results indicate that increasing the support point number provides a viable strategy for the realization of an ultra-low thermal sensitivity mirror assembly which retains high support stiffness.

## Figures and Tables

**Table 1:** Support member radial stiffness factor for multi-point support mode.

	$K_{R3}$	$K_{R4}$	$K_{R5}$	$K_{R6}$	$K_{R7}$	$K_{R8}$
Multiplier	$\frac{1}{1.5}$	$\frac{1}{2}$	$\frac{1}{2.5}$	$\frac{1}{3}$	$\frac{1}{3.5}$	$\frac{1}{4}$

**Table 2:** Requirements of the primary mirror for an AOIS.

Parameters		Value
Aperture size	Outer diameter	260 mm
	Central perforation diameter	80 mm
Surface displacement due to gravity		≤1 micron
Surface deformation RMS due to 35 °C temperature variation		≤λ/35(λ = 632.8 nm)

**Table 3:** Material properties of the mirror assembly.

Material	Density *ρ* (g/cm^3^)	CTE α (×10^−6^)	Young’s Modulus *E* (GPa)	Poisson’s Ratio ν
Zerodur	2.53	0 ± 0.05	90.6	0.24
Ti-6Al-4V	4.44	8.9	114	0.34
Invar	8.13	0.31	148	0.29

## Data Availability

Not applicable.
